# Medical student‐led simulation in COVID‐19 crisis: foundation doctor perspectives

**DOI:** 10.1111/tct.13344

**Published:** 2021-03-28

**Authors:** Prasanti Alekhya Kotta, Rama Lakshman

**Affiliations:** ^1^ Department of Medicine St Thomas’ Hospital London UK; ^2^ Department of Medicine King’s College Hospital London UK

We read with interest the article by Ekert et al and commend their efforts on organising medical student‐led simulation during COVID‐19.^1^ As two newly qualified doctors, we experienced first‐hand the challenges posed by the pandemic, including being redeployed to unfamiliar settings and confronting suffering and death regularly. Simulation is an excellent way to increase confidence and overcome anxiety,^2^ and using medical students to facilitate simulation is very appropriate for a novel disease like COVID‐19, which negates the usual hierarchies of clinical experience.

Having organised near‐peer simulation during medical school, we can attest to the positive experiences of the student facilitators. Clinical scenarios we ran in simulation have become those we are most comfortable managing as doctors. Students may have found it daunting to take a leadership role amongst more experienced health care professionals, but this is a common occurrence for newly qualified doctors. Running simulation is a valuable opportunity to interact with the multidisciplinary team and understand the skills they provide.

We participated in COVID‐19 simulation ourselves and benefited from practicing clinical scenarios in a safe environment, learning about management guidelines and good clinical practices such as advanced care planning.

Social distancing during the pandemic could prevent simulation being offered to everyone who could benefit. One potential solution is virtual simulation: videoconferencing platforms, for example, Zoom can be utilised to live‐stream scenarios to participants and features such as ‘breakout rooms’ can be used to achieve small group interactivity. Recent research shows that virtual simulation provides safe and effective training.^3^

## FUNDING

The authors have no sources of funding or disclosures.

## CONFLICT OF INTEREST

The authors have no conflict of interest.
